# Primary and mental health service use in community health center patients before and after cancer diagnosis

**DOI:** 10.1002/cam4.4524

**Published:** 2022-04-28

**Authors:** Annie E. Larson, Heather Angier, Andrew Suchocki, Robert W. Voss, Miguel Marino, Nathaniel Warren, Nathalie Huguet

**Affiliations:** ^1^ Research Department OCHIN Inc. Portland Oregon USA; ^2^ Department of Family Medicine Oregon Health & Science University Portland Oregon USA; ^3^ Clackamas Health Centers Oregon City Oregon USA; ^4^ Biostatistics Group Oregon Health & Science University Portland Oregon USA

**Keywords:** cancer survivors, community health centers, health care disparities, preventive services, utilization of health services

## Abstract

**Background:**

Cancer survivors face increased risk for chronic diseases resulting from cancer, preexisting conditions, and cancer treatment. Having an established primary care clinic or health insurance may influence patients’ receipt of recommended preventive care necessary to manage, treat, or diagnose new conditions. This study sought to understand receipt of healthcare in community health centers (CHCs) before and after cancer diagnosis among cancer survivors. We also examined the type of care received and assessed whether being established with a CHC or the type of health insurance affected the use of services.

**Methods:**

Using electronic health record data and linked cancer registries from 5,649 CHC patients in three states from 2012 through 2018, we obtained monthly rates of primary care and mental health/behavioral health (MHBH) visits and the probability of receipt of care before and after a cancer diagnosis.

**Results:**

Seventy‐five percent of CHC patients diagnosed with cancer returned to their primary CHC for care within 2‐years of their diagnosis. Among those who returned, there was a sharp increase in primary and MHBH care shortly before their diagnosis. Significantly more primary care (pre: 19.6%, post: 21.9%, *p* < 0.001) and MHBH care (pre: 1.2%, post: 1.6%, *p* < 0.001) was received after diagnosis than before. However, uninsured patients had fewer visits after their diagnosis than before.

**Conclusion:**

Use of preventive care for cancer survivors is particularly important. Having an established primary care clinic may help to ensure survivors receive recommended screening and care.

## Graphical abstract

In this study on the receipt of preventive healthcare among community health center patients before and after a cancer diagnosis, we found that significantly more primary and mental healthcare was received after diagnosis than before. However, uninsured patients had fewer visits after their diagnosis than before.

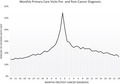

## 1 INTRODUCTION

The National Coalition for Cancer Survivors defines cancer survivorship as the time from diagnosis through treatment and beyond.^1^ As treatment options for cancer are improving, the number of cancer survivors has increased and 5‐year survival rates are now more than 90% for some types of cancer.^2^ The number of cancer survivors in the US is estimated to increase from 15.5 million to 26.1 million by 2040.^3^

As cancer survival has improved, survivors face increased risk for chronic diseases.^3^, ^4^ This increased risk comes from the cancer itself, other preexisting conditions, and cancer treatment, which can result in accelerated aging including cardiovascular impairments.^5^, ^6^ Continued cancer screening, monitoring, and ongoing general healthcare services to manage, treat, or diagnose new conditions is important for survivors. Additionally, cancer diagnosis has been associated with a higher rate of mental health conditions than patients without a history of cancer.^7^, ^8^, ^9^

Despite the importance of preventive and primary care utilization, disparities in access and use of healthcare exist among cancer survivors. Cancer survivors with health insurance,^10^, ^11^ lower out of pocket costs,^12^ and greater continuity of care are more likely to utilize preventive cancer screenings after a cancer diagnosis than their counterparts.^13^ Further research suggests that a well‐established relationship with a primary care clinic or clinician may be the most important factor to ensure recommended care is received during the transition period from active cancer treatment for survivors.^14^ Some suggest survivors may experience a “fear of recurrence” resulting in an increased desire for surveillance and testing.^15^ However, evidence regarding preventive and primary care use among survivors is often conflicting.^16^, ^17^

Community health centers (CHCs) provide care to the US safety net population, many of whom do not have health insurance or may not be able to afford care elsewhere. CHCs help uninsured and underinsured patients navigate the healthcare system, ensuring equitable access to preventive services. Despite the fact that patients seen in CHCs have lower income and are at greater risk for poor health outcomes, patients served in CHCs are more likely to utilize preventive services compared to those cared for in other ambulatory primary care settings.^18^ However, it is unclear if this is true with respect to cancer survivors. While most providers in CHCs believe primary care should play a role in survivorship care, only 20% of CHC providers felt confident in their ability to fully care for survivors compared to roughly 40% of providers from a national sample.^19^

This study sought to understand receipt of healthcare in CHCs before and after cancer diagnosis among cancer survivors. We sought to examine the type of care received and assessed whether being established with a CHC or the type of health insurance affected the use of services.

## 2 DATA AND METHODS

### 2.1 Data sources

We utilized electronic health record (EHR) data from OCHIN’s data warehouse, a part of the Accelerating Data Value Across a National Community Health Center (ADVANCE) Clinical Research Network, a member of PCORnet.^20^ OCHIN provides a fully hosted, single instance of the Epic© EHR to CHCs across 19 states. EHR data are centrally collected and standardized resulting in a robust longitudinal repository of clinical data for patients seen in member CHCs.^20^ The demographic profile of patients in OCHIN CHCs mirrors that of national estimates from all CHCs.^21^ We analyzed visits from 2012 through 2018 to primary care clinics and local health departments that were “live” on OCHIN’s EHR by 1/1/2013 and live for at least 3‐years. Additionally, we utilized linked cancer registry data from Oregon, Washington, and California through 12/31/2017.^22^ These states were selected because OCHIN has data use agreements with them. The additional year of data from the EHR allowed for patients who were diagnosed with cancer at the end of 2017 at least 1 year to return for care in a CHC.

### 2.2 Ethics statement

This study was approved by the Oregon Health & Science University institutional review board.

### 2.3 Study population

Our dataset was limited to patients 19–64 years of age at least 1 day during the study period, 1/1/2012–12/31/2018, who had at least one primary care visit at an eligible CHC (defined above) within 2 years prior to their cancer diagnosis. Patients were considered diagnosed with cancer if they were included in one of the linked state cancer registries. Analyses on patients with visits to CHCs before and after their diagnosis were further restricted to those with at least one visit within 2 years prior to and one visit within 2 years after their cancer diagnosis.

### 2.4 Outcomes

Our outcomes included primary care and mental health/behavioral health (MHBH) visits. We utilized Meaningful Use‐qualifying CPT codes from patient encounters to determine whether a patient had a visit in a given month during the 2 years before and 2 years after their cancer diagnosis. We created a balanced person‐month panel dataset for the 4‐year study period. Each person‐month included information about whether or not primary and/or MHBH care was received. For person‐months without encounter data, it was assumed the patient did not receive any care.

Additionally, we examined utilization of several US Preventive Service Task Force (USPSTF) A or B recommended preventive screenings^23^ including: blood pressure (BP) screening for all study‐eligible patients at each encounter, patient health questionnaire (PHQ) screening for depression using either the PHQ‐2 or PHQ‐9 questionnaire for all study‐eligible patients, and hemoglobin A1c (A1c) screening among patients with diabetes. These measures were selected to represent several categories of healthcare representing physical health screenings (BP), mental health screenings (PHQ), and chronic disease management (A1c) and were used as a proxy to measure quality of care. We created a dataset with a binary indicator for ever having received each of these services within the 2 year pre‐ and post‐cancer diagnosis periods.

### 2.5 Covariates

Covariates of interest included: (1) a binary indicator for encounters before or after a patient's cancer diagnosis, (2) length of established care with the patients' primary care clinic, and (3) health insurance type. Length of established care was defined by identifying the CHC each patient was most frequently seen at in the 2‐years prior to their cancer diagnosis. If a patient received care at more than one CHC with equal frequency, we used the CHC they were seen at closest to their cancer diagnosis. We then identified the earliest visit date at their primary CHC and calculated the length of time from that visit to their first cancer diagnosis. Health insurance type was defined using health insurance types billed during visits in the 2‐years prior to diagnosis. We defined patients with all uninsured visits as continuously uninsured, those with all visits insured by any type of public insurance as continuously publicly insured, and those with all visits paid by private insurance as continuously privately insured. For patients who changed from one type of insurance to another, they were defined as having discontinuous insurance type. Patients who changed from being uninsured to insured or vice versa were defined as episodically insured. For analyses using the person‐month dataset, we also examined time from cancer diagnosis calculated as months from the first cancer diagnosis to the month of each visit.

Our models adjusted for age at cancer diagnosis, race (White, Black, other race, unknown race), ethnicity (Hispanic, non‐Hispanic, unknown ethnicity), sex, rurality (urban, suburban, large rural, small rural), and stage of cancer at diagnosis (stage 0: in situ, stage 1: localized, stage 2–3: regional, stage 4–5: regional, stage 7: distant, missing/unassigned).

### 2.6 Statistical analysis

We described our patients, comparing those who had a visit to a CHC before and after their cancer diagnosis to those who only had a visit prior to their cancer diagnosis using chi‐square tests and *t*‐tests to assess statistical differences between groups. All additional analyses were restricted to patients who had at least one visit before and at least one visit after their cancer diagnosis. We examined monthly trends in the frequency of having received a primary care or MHBH visit in the 2 years before and after cancer diagnosis. Using the balanced patient‐month dataset, we estimated the effect of our covariates of interest on the predicted probability of having a primary care visit or MHBH visit in a given month using logistic regression models and estimated marginal effects, clustering on the patient.

Using the dataset with a binary indicator for ever receiving each service type we examined receipt of USPSTF recommended service in the 2 years before or after a patient's cancer diagnosis. We used logistic regression models to estimate the predicted probability of receiving care. Additionally, to evaluate the impact of the length of established care and health insurance type on receipt of USPSTF recommended screening before and after a cancer diagnosis we used the logistic regression models mentioned above and included an interaction term for length of established care at a CHC and the pre‐/post‐diagnosis indicator.

This study used Stata 15.1 for all statistical analyses.

## 3 RESULTS

There were 5,649 patients included in our study population, 4,179 (74.0%) who returned for care at a CHC within 2 years of their cancer diagnosis and 1,470 (26.0%) who had not returned for care. Patients who returned to a CHC for care after a cancer diagnosis were more likely to be female, White, non‐Hispanic, and be publicly insured or episodically insured than patients who did not return to a CHC within 2 years of their cancer diagnosis (Table 1). Those who returned had significantly more visits to a CHC prior to their diagnosis, were more likely to live in a suburban or large rural area and were less likely to have an advanced stage of cancer at diagnosis. Additionally, returning patients were established at their primary CHC for significantly longer than those who did not return. Of those who returned, 86.1% had the same primary CHC post‐diagnosis that they used pre‐diagnosis.

**TABLE 1:** Demographics of cancer survivors in CHCs: returning patients versus non‐returning patients

	Total study population (*N* = 5,649)	Visits before & after cancer Dx (*N* = 4,179)	Visits before, not after cancer Dx (*N* = 1,470)	*p*‐value (before & after vs. before only)
Median # visits (annually)	5.1	5.9	3.2	<0.001
Avg. age at diagnosis	52.3	52.2	52.6	0.265
Sex				0.001
Male	40.4%	39.1%	44.2%	
Female	59.6%	60.9%	55.9%	
Race				0.001
White	86.1%	86.7%	84.2%	
Black	4.7%	4.3%	5.9%	
Other race	5.4%	5.6%	4.9%	
Unknown race	3.8%	3.4%	5.0%	
Ethnicity				<0.001
Hispanic	16.9%	17.2%	16.1%	
Non‐Hispanic	79.6%	80.1%	78.3%	
Unknown ethnicity	3.5%	2.8%	5.6%	
Insurance before cancer Dx				<0.001
Cont. private/marketplace	9.5%	9.3%	10.0%	
Cont. public	41.7%	43.2%	37.2%	
Cont. uninsured	16.2%	12.9%	25.8%	
Discont. insurance type	3.4%	3.6%	2.9%	
Episodically insured	29.2%	30.9%	24.2%	
Rurality at cancer diagnosis				0.006
Urban	61.1%	60.2%	63.6%	
Suburban	9.0%	9.1%	8.8%	
Large rural	22.0%	23.1%	19.1%	
Small rural	7.5%	7.2%	8.4%	
Unknown	0.4%	<1%	<1%	
Stage at diagnosis				<0.001
Stage 0: in situ	9.2%	10.1%	6.7%	
Stage 1: localized	39.9%	42.3%	32.9%	
Stage 2–3: regional	16.1%	16.4%	15.4%	
Stage 4–5: regional	5.8%	5.6%	6.7%	
Stage 7: distant	16.1%	13.6%	23.3%	
Missing/unassigned stage	12.8%	12.1%	14.9%	
Length of CHC engagement				
Avg. yrs. seen at primary CHC (s.d.)	2.4 (2.3)	2.5 (2.3)	2.3 (2.1)	0.010
Use same CHC pre as post	63.7%	86.1%	N/A	

**FIGURE 1:**
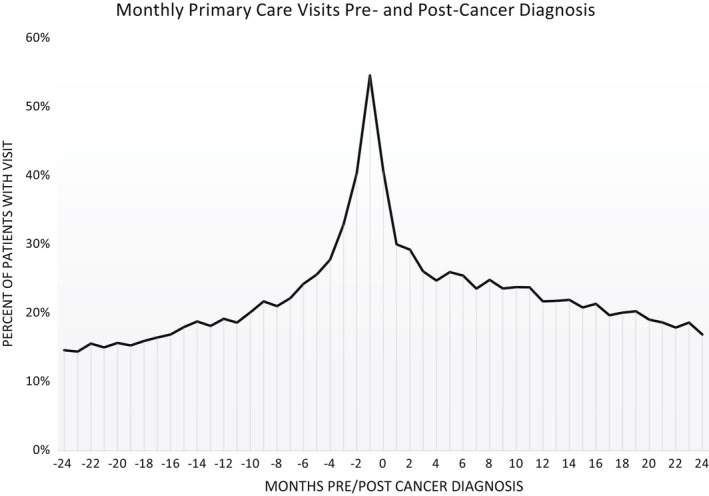
Monthly primary care visits pre‐ and post‐cancer diagnosis. percent of cancer survivors with a primary care visit in a given month 2‐years before and 2‐years after their cancer diagnosis

**FIGURE 2:**
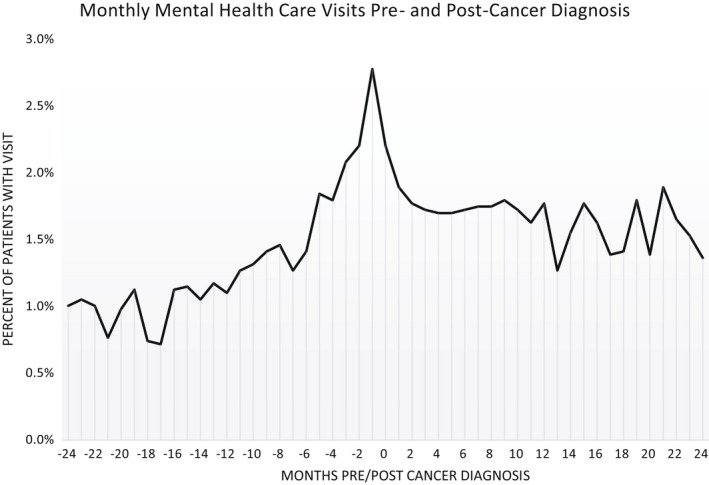
Monthly mental health care visits pre‐ and post‐cancer diagnosis. percent of cancer survivors with a mental health / behavioral health (MHBH) visit in a given month 2‐years before and 2‐years after their cancer diagnosis

In the 2 years leading up to a cancer diagnosis, there was a steady increase in primary care visits, with the peak in the month prior to their diagnosis where almost 55% of eligible patients had at least one primary care visit (Figure 1). This was followed by a steady decline in primary care visits in the 2 years after diagnosis. However, the percent of patients with a visit post‐diagnosis remained higher than prior to their cancer diagnosis. While the percentage of patients with MHBH care visits was much lower than it was for primary care, we saw a similar trend before and after a patients' cancer diagnosis (Figure 2).

Estimates from the person‐month panel dataset logistic regression models found the predicted probability of a monthly primary care visit increased significantly from 19.6% pre‐diagnosis (95% CI: 19.2, 20.0) to 21.9% post‐diagnosis (95% CI: 21.4, 22.5, Table 2). Those with longer lengths of established care at a CHC were more likely to have a primary care visit pre‐ and post‐cancer diagnosis. However, there was a smaller difference in the predicted probability of a primary care visit post‐diagnosis by length of a CHC engagement (5.5 percentage points) than pre‐diagnosis (17.3 percentage points). Uninsured and privately insured patients were significantly less likely to have a primary care visit than all other insurance types both before and after a cancer diagnosis. While the likelihood of a primary care visit increased for those publicly insured (from 20.8% to 22.7%, *p *< 0.001), those with discontinuous insurance types (from 19.2% to 25.3%, *p *< 0.001), and those who were episodically insured (from 19.7% to 22.1%, *p *< 0.001), it decreased for those who were uninsured (from 16.0% to 11.7%, *p* < 0.001). Similar to the use of primary care, MHBH visits increased significantly from pre‐ to post‐cancer diagnosis (from 1.2% to 1.6%, *p* < 0.001). Receipt of MHBH was significantly greater for patient's pre‐cancer diagnosis among those established in their CHC more than 5 years compared to those established <1 year (1.7% vs. 0.6%, *p* < 0.001), but did not significantly differ by length of established care post‐diagnosis.

**TABLE 2:** Predicted probability of monthly visit 2 years before and after cancer diagnosis

	Pred. prob. of visit before cancer diagnosis	95% CI	Pred. prob. of visit after cancer diagnosis	95% CI
**Primary care visit**				
Pre‐/post‐diagnosis	19.6%	(19.2, 20.0)	21.9%	(21.4, 22.5)
Length of CHC engagement prior to cancer diagnosis				
<1 year	9.3%	(8.9, 9.6)	19.8%	(18.9, 20.6)
1–3 years	24.2%	(23.2, 24.9)	22.0%	(21.0, 22.9)
3–5 years	24.7%	(23.4, 25.7)	22.8%	(21.5, 24.1)
5+ years	26.9%	(25.7, 28.1)	25.3%	(24.0, 26.6)
Time from cancer diagnosis				
1 month from cancer diagnosis	28.4%	(27.7, 29.1)	28.9%	(28.2, 29.7)
1 year from cancer diagnosis	19.2%	(18.8, 19.6)	21.7%	(21.1, 22.2)
2 years from cancer diagnosis	12.4%	(11.8, 12.9)	15.8%	(15.2, 16.5)
Insurance				
Cont. uninsured	16.0%	(14.4, 17.6)	11.7%	(10.0, 13.4)
Cont. publicly insured	20.8%	(20.1, 21.5)	22.7%	(21.8, 23.6)
Cont. privately/marketplace insured	14.6%	(13.1, 16.0)	15.2%	(13.5, 16.9)
Discontinuous insurance type	19.2%	(18.2, 20.3)	25.3%	(23.7, 26.8)
Episodically insured	19.7%	(19.0, 20.3)	22.1%	(21.2, 22.9)
**Mental health/behavioral health care visit**				
Pre‐/post‐diagnosis	1.2%	(1.0, 1.3)	1.6%	(1.4, 1.8)
Length of CHC engagement prior to cancer diagnosis				
<1 year	0.6%	(0.4, 0.7)	1.6%	(1.3, 1.9)
1–3 years	1.3%	(1.0, 1.6)	1.5%	(1.2, 1.8)
3–5 years	1.7%	(1.2, 2.2)	1.9%	(1.3, 2.4)
5+ years	1.7%	(1.2, 2.2)	1.4%	(1.0, 1.8)
Time from cancer diagnosis				
1 month from cancer diagnosis	1.7%	(1.5, 2.0)	1.8%	(1.5, 2.0)
1 year from cancer diagnosis	1.2%	(1.0, 1.4)	1.6%	(1.4, 1.8)
2 years from cancer diagnosis	0.8%	(0.6, 1.0)	1.4%	(1.1, 1.7)
Insurance				
Cont. uninsured	1.3%	(0.6, 2.0)	1.4%	(0.4, 1.8)
Cont. publicly insured	1.1%	(0.8, 1.3)	1.4%ṇ	(1.1, 1.6)
Cont. privately/marketplace insured	0.1%	(0.01, 0.3)	0.1%	(0.01, 0.2)
Discontinuous insurance type	0.8%	(0.5, 1.2)	1.8%	(1.3, 2.3)
Episodically insured	1.5%	(1.2, 1.8)	1.9%	(1.6, 2.3)

Note

Adjusts for age at diagnosis, stage of cancer at diagnosis, race, ethnicity, sex, and rurality.

We found significant differences in USPSTF recommended screenings before and after cancer diagnosis (Table 3). There was a significant increase in the predicted probability of ever having a PHQ screening among all patients (from 75.9% to 89.5%, *p* < 0.001) and A1c screening among the 1,075 cancer survivors with diabetes (from 79.2% to 98.4%, *p* < 0.001) from pre‐ to post‐cancer. Conversely, we found a decrease in the predicted probability of BP screenings at each encounter among all patients (from 99.4% to 95.3%, *p* < 0.001). Patients who had established care at their primary CHC for five or more years were 3 percentage points more likely to receive BP screenings at an encounter than those with established care for less than 1 year (*p* < 0.001). There was a 10 percentage point difference between diabetics with more than 5‐years of care received at their primary CHC as compared to those who were established for less than 1 year. Patients who had established care for ≥5 years were less likely to receive a PHQ screening than those established <1 year (79.8% vs. 84.3%, respectively, *p *< 0.001). While length of time the patient had established care for was associated with receipt of USPSTF recommended screenings, it did not have significantly different effects on use of PHQ, A1c, or BP screenings before as compared to after cancer diagnosis (results not shown).

**TABLE 3:** Predicted probability of preventive service use among cancer survivors

	Pred. prob.	95% CI
**BP screening at each encounter**		
Pre‐/post‐cancer diagnosis		
Pre‐diagnosis	99.4%	(99.1, 99.6)
Post‐diagnosis	95.3%	(94.6, 95.9)
Length of CHC engagement		
<1 year	96.4%	(95.8, 97.1)
1–3 years	97.1%	(96.5, 97.8)
3–5 years	97.4%	(96.6, 98.2)
5+ years	99.4%	(99.0, 99.8)
**PHQ screening**		
Pre‐/post‐cancer diagnosis		
Pre‐diagnosis	75.9%	(74.6, 77.2)
Post‐diagnosis	89.5%	(88.6, 90.4)
Length of CHC engagement		
<1 year	84.3%	(83.0, 85.6)
1–3 years	83.3%	(81.9, 84.7)
3–5 years	81.3%	(79.3, 83.3)
5+ years	79.8%	(77.7, 81.9)
**A1c screening—diabetics**		
Pre‐/post‐cancer diagnosis		
Pre‐diagnosis	79.2%	(76.8, 81.5)
Post‐diagnosis	98.4%	(97.7, 99.2)
Length of CHC engagement		
<1 year	82.7%	(79.8, 88.6)
1–3 years	88.7%	(86.4, 91.0)
3–5 years	93.4%	(91.1, 95.7)
5+ years	92.7%	(90.4, 90.8)

Note

Adjusts for age at cancer diagnosis, stage of cancer at diagnosis, race, ethnicity, rurality, and insurance.

## 4 DISCUSSION

Most patients who received care in a CHC prior to their cancer diagnosis returned to a CHC for primary care and/or MHBH care within 2‐years of their diagnosis and most returned to the same CHC. Unsurprisingly, these patients were less likely to have an advanced stage of cancer. However, the fact that those who were continuously uninsured were less likely to return for care after their diagnosis is concerning. While it is possible that they may have received care outside the OCHIN network, it is more likely that they stopped receiving primary care altogether. The Affordable Care Act (ACA) expanded Medicaid eligibility in many states and provided subsidies to purchase health insurance through federal and state marketplaces to those who did not qualify for Medicaid.^24^ This led to a decrease in uninsurance for many cancer survivors^25^ and may have resulted in some previously uninsured patients seeking care outside CHCs. Yet, 1 in 5 cancer survivors remain uninsured in states that did not expand Medicaid.^25^

Among cancer survivors who returned to a CHC for care after their diagnosis, we saw a large increase in primary care and MHBH care visits leading up their diagnosis, with the highest rate occurring 1 month prior to diagnosis. This mirrors findings from a previous study examining Danish cancer survivors.^26^ The steady increase in primary and MHBH care leading up to a cancer diagnosis suggests awareness of concerning health issues. Further, this increase in care occurred shortly before cancer diagnosis indicating CHC providers were likely appropriately screening patients. We found that cancer survivors who returned to a CHC for care had significantly more primary care and MHBH care visits post‐diagnosis compared to pre‐diagnosis. Additionally, we saw increase in PHQ screening among all patients and A1c screening among patients with diabetes from before to after their cancer diagnosis. Interestingly, we found a decline in BP screenings at eligible patient encounters post‐diagnosis.

While patients who were more established at their CHC were more likely to receive both BP and A1c screenings as recommended, these patients were less likely to receive PHQ screenings than those less established. This could be due to the fact that USPSTF recommendations for PHQ screening are vague about the timing and interval screening should occur^27^ potentially resulting in screening only happening for new patients. Additionally, clinics may have defined their own PHQ screening intervals for established patients.

Although we did not have information on care received by an oncologist, previous research shows that patients receiving care from only a clinician were more likely to receive recommended preventive care than those who only received care from an oncologist.^28^ Lack of clarity and coordination of roles in the transition of survivorship care may result in missed recommended preventive screenings. The relationship with one's clinician has been found to be particularly important in the transition to or from specialty care.^14^ While CHC clinicians prefer a shared care model for care of survivors, previous research found they feel limited by lack of training, and poor communication with oncologists.^19^, ^29^ As more cancer survivors transition back to primary care, it is critical to ensure clinicians have the necessary training to care for survivors beyond recommended screenings, preventive, and MHBH healthcare. More research is needed to identify optimal strategies to educate clinician in survivorship care and opportunities to improve care coordination and communication between specialists and clinicians to ensure appropriate survivorship care.

### 4.1 Limitations

The use of EHR data limits us from knowing whether patients received care outside a CHC. However, previous research found most patients continue to receive care in CHCs over time and attrition rates in CHCs are similar to those in studies using prospectively collected data.^30^ We did not account for death as we had limited information available and there were discrepancies between the EHR and cancer registry data. Where date of death was available, less than 1% of study patients died during the study period. We recognize that this is most like an underestimate of the true number of deaths.^31^ As such we are unable to account for how survival impacts our results. Finally, we recognize the ACA resulted in reduced out of pocket costs for many preventive services The present analysis did not account for those changes which went into effect in 2014.^24^

## 5 CONCLUSION

Receipt of recommended preventive care is important for cancer survivors. We found that most cancer survivors return to their primary CHC for care after their diagnosis to receive primary care and MHBH care. Those with more established relationships with their primary CHC received more preventive and MHBH care both before and after their cancer diagnosis. Of concern is that uninsured cancer survivors received less preventive and MHBH care after a cancer diagnosis. Thus, it will be important to ensure these patients are continuing to receive needed and recommended care.

## CONFLICT OF INTEREST

The authors of this paper have no conflict of interest to report.

## AUTHOR CONTRIBUTIONS

Annie E Larson: Conceptualization, supervision, formal analysis, visualization, and writing—original draft preparation. Heather Angier: Conceptualization, visualization, writing—review, and editing. Andrew Suchocki: Conceptualization, visualization, writing—review, and editing. Robert W. Voss: Data curation, writing—review, and editing. Miguel Marino: Methodology, writing—review, and editing. Nathanial Warren: Project administration, writing—review, and editing. Nathalie Huguet: Supervision, writing—review, and editing.

## DISCLAIMERS

The research presented in this paper is that of the authors and does not reflect that of the California Department of Public Health nor the views of the funding agencies.

## ETHICAL APPROVAL

This study was approved by the Oregon Health & Science University institutional review board.

## Data Availability

Raw data underlying this article were generated from multiple agencies and institutions; restrictions apply to the availability and re‐release of data under cross‐institution agreements. Data are however available from the authors upon reasonable request and with permission of all relevant parties.
